# Small RNA sequencing of extracellular vesicles identifies circulating miRNAs related to inflammation and oxidative stress in HIV patients

**DOI:** 10.1186/s12865-020-00386-5

**Published:** 2020-11-11

**Authors:** Sukrutha Chettimada, David R. Lorenz, Vikas Misra, Steven M. Wolinsky, Dana Gabuzda

**Affiliations:** 1 Department of Cancer Immunology and Virology, Dana-Farber Cancer Institute, Boston, MA USA; 2 Division of Infectious Diseases, Department of Medicine, Northwestern University Feinberg School of Medicine, Chicago, IL USA; 3 Department of Neurology, Harvard Medical School, Boston, MA USA

**Keywords:** Small RNA, MiRNA, Extracellular vesicles, Exosomes, HIV, Inflammation, Oxidative stress

## Abstract

**Background:**

Extracellular vesicles (EVs) are nano-sized particles secreted by most cells. EVs carry nucleic acids that hold promise as potential biomarkers in various diseases. Human immunodeficiency virus type 1 (HIV) infects CD4+ T cells and induces immune dysfunction, inflammation, and EV secretion, but little is known about EV small RNA cargo in relation to immune dysregulation in HIV-infected individuals. Here, we characterize small RNA carried by circulating EVs in HIV-positive subjects on antiretroviral therapy (ART) relative to uninfected controls by next-generation RNA sequencing.

**Results:**

Plasma EVs isolated from HIV-positive and HIV-negative subjects in test (*n* = 24) and validation (*n* = 16) cohorts were characterized by electron microscopy, nanoparticle tracking analysis, and immunoblotting for exosome markers. EVs were more abundant in plasma from HIV-positive compared to HIV-negative subjects. Small RNA sequencing of plasma EVs in the test cohort identified diverse small RNA species including miRNA, piRNA, snRNA, snoRNA, tRNA, and rRNA, with miRNA being the most abundant. A total of 351 different miRNAs were detected in plasma EVs, with the top 50 miRNAs accounting for 90% of all miRNA reads. miR-26a-5p was the most abundant miRNA, followed by miR-21-5p and miR-148-3p. qRT-PCR analysis showed that six miRNAs (miR-10a-5p, − 21-5p, −27b-3p, − 122-5p, −146a-5p, − 423-5p) were significantly increased in plasma EVs from HIV-positive compared to HIV-negative subjects in the validation cohort. Furthermore, miR-21-5p, −27b-3p, −146a-5p, and − 423-5p correlated positively with metabolite markers of oxidative stress and negatively with anti-inflammatory polyunsaturated fatty acids. Over-representation and pathway enrichment analyses of miRNAs and their target genes predicted functional association with oxidative stress responses, interferon gamma signaling, Toll-like receptor signaling, TGF beta signaling, and Notch signaling.

**Conclusions:**

HIV-positive individuals on ART have increased abundance of circulating EVs carrying diverse small RNAs, with miRNAs being the most abundant. Several miRNAs associated with inflammation and oxidative stress are increased in circulating EVs of HIV-positive individuals, representing potential biomarkers of targetable pathways that contribute to disease pathogenesis.

**Supplementary Information:**

The online version contains supplementary material available at 10.1186/s12865-020-00386-5.

## Background

Human immunodeficiency virus type 1 (HIV) infection is characterized by progressive decline of CD4+ T cell counts, increased immune activation, and inflammation. Although antiretroviral therapy (ART) increases CD4+ T cell counts and improves overall health and life expectancy of HIV-positive individuals, immune activation and chronic inflammation persist. Causes of chronic inflammation in ART-treated HIV patients are incompletely understood, but likely include microbial translocation, elevated expression of type I and II interferons, altered chemokine and cytokine production, and co-infections (e.g., Hepatitis C virus (HCV)) [1–3]. HIV infection and chronic inflammation contribute to generation and accumulation of reactive oxygen species (ROS), compromising antioxidant pathways and leading to oxidative stress, a predictor of morbidity and mortality [4–6]. Identification of biomarkers associated with HIV pathogenesis, inflammation, and oxidative stress is important to gain insights into underlying mechanisms and discover prognostic and diagnostic markers.

Extracellular vesicles (EVs) and their protein and nucleic acid cargo have been extensively studied and used as biomarkers in various diseases including HIV, cancer, cardiovascular diseases, and neurological disorders [7]. EVs, including exosomes (30–150 nm), microvesicles (MV; microparticles) (100 nm–1 μm), and apoptotic bodies (> 1 μm), are secreted by most cell types and have been isolated from plasma and other body fluids. EV cargo includes proteins, lipids, mRNAs, long non-coding RNAs (lncRNA), and several species of small non-coding RNA, such as microRNA (miRNA), Piwi-interacting RNA (piRNA), small nucleolar RNA (snoRNAs), and small nuclear RNA (snRNA), or RNA fragments [8], and provide a means for transfer of RNA between donor and recipient cells. Specific cargo of EVs is dependent on the cell of origin as well as biological conditions such as infection, inflammation, and stress. EVs are involved in cell-to-cell communication and are proposed to play a role in maintaining homeostasis; they have also been implicated in spreading infections via transport of viral and microbial products [7]. EVs, particularly exosomes, can also regulate gene expression by transporting miRNAs to recipient cells and post-transcriptionally controlling translation of corresponding miRNA targets in recipient cells, thereby affecting cellular responses to stress, inflammation, and cell death [9].

Previous studies have shown that HIV-positive individuals have higher abundance of circulating EVs compared to healthy controls [10–12]. EV protein cargo in HIV infection has been studied, revealing enrichment of HIV virulence factors and pro-inflammatory cytokines and chemokines [10, 13–16]. HIV infection induces EV secretion, and EVs from HIV-infected cells transport viral and host components that promote spreading of infection [13–15]. EVs can also inhibit HIV infection by carrying protective factors such as APOBEC3 and interferons [17, 18]. Additionally, EV RNA exhibiting 5′-triphosphate ends stimulates RIG-I, which induces an interferon response [19]. We previously showed that plasma EVs in ART-treated HIV-positive individuals carry proteins related to immune activation and oxidative stress, and have immunomodulatory effects on myeloid cells, suggesting functional links to inflammation and redox pathways during pathogenesis [12]. Limited studies have explored miRNA content of EVs, showing enrichment of specific miRNAs associated with inflammation and fatal liver disease in HIV-positive individuals [11, 20], or lower neuropsychological performance [21]. Little is known about the small RNA repertoire of circulating EVs in HIV-positive individuals. Given that small RNAs are enriched in EVs, we characterized small RNA cargo of plasma EVs in HIV-positive individuals on ART relative to uninfected controls by small RNA sequencing. We then validated some of the mapped miRNAs that are upregulated in treated HIV disease in an independent cohort and examined their association with metabolite markers and pathways related to inflammation and oxidative stress.

## Results

### Characteristics of the study cohort

**Fig. 1:**
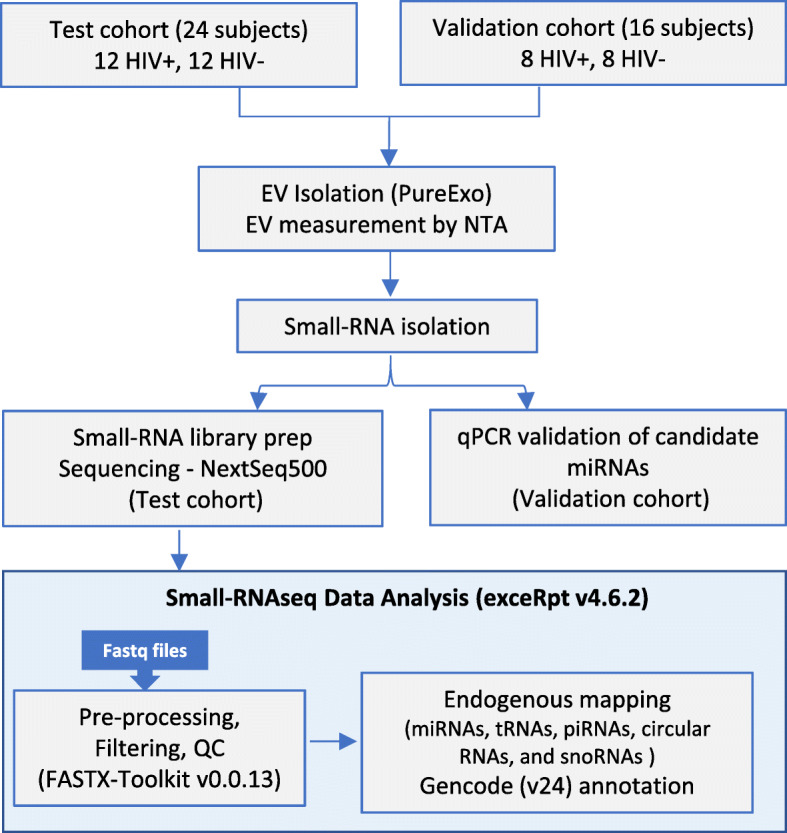
Study design summarizing the cohort, methodology, and data analysis. 500 μl plasma from subjects in test or validation cohorts were used to isolate EVs using the PureExo kit. EVs were measured by nanoparticle tracking analysis followed by small RNA isolation. Small RNA libraries were prepared and sequenced on Nextseq500, and data was analyzed using the exceRpt pipeline. Candidate miRNAs were validated by qPCR

A total of 24 subjects (12 HIV-positive and 12 HIV-negative) and 16 subjects (8 HIV-positive and 8 HIV-negative) subjects comprised the test and validation cohorts, respectively (Fig. 1). Demographic and clinical characteristics are summarized in Table 1. The median age across cohorts was 54 [interquartile range (IQR): 48, 50]. The test cohort was comprised of all males (63% black), while the validation cohort was 63% male (38% black). All HIV-positive subjects were on ART with suppressed or low plasma viral load. Compared to the test cohort, HIV-positive subjects in the validation cohort had lower median CD4 T cell counts, CD4 nadir, CD4:CD8 ratio, and longer median duration of HIV infection, indicating more advanced HIV disease. Both cohorts had high prevalence of smoking (54 and 56% in test and validation cohorts, respectively) and cocaine use (50% in both cohorts). HIV-negative and HIV-positive groups within each cohort were balanced with respect to age, race, HCV status, and cocaine use. Two subjects in the test cohort and no subjects in the validation cohort were HCV-seropositive. All subjects in both cohorts were negative for Hepatitis B virus (HBV) surface antigen and/or DNA.

**Table 1:** Clinical and demographic characteristics of test and validation cohorts

	Test cohort		Validation cohort	
	HIV-negative (***n*** = 12)	HIV-positive (***n*** = 12)	HIV-negative (***n*** = 8)	HIV-positive (***n*** = 8)
Age^α^	52 [44–59]	55 [45–60]	53 [51–54]	57 [53–61]
Black race	7 (58)	8 (67)	3 (38)	3 (38)
Male gender	12 (100)	12 (100)	3 (38)	7 (88)
Duration of HIV infection (years)^α^		13 [11–20]		19 [13–22]
Viral load (copies/ml)		40 [10–80]		40 [40–40]
Viral load < 200 copies/ml		11 (92)		8 (100)
CD4 count (cells/μl)	955 [776–1142]	619 [430–734]		382 [285–495]
CD4 nadir (cells/μl)	639 [539–796]	166 [92–257]		82 [26–221]
CD4/CD8 ratio^α^	1.4 [1.1–1.9]	0.73 [0.54–1.0]		0.41 [0.26–0.51]
ART use		12 (100)		8 (100)
Protease inhibitor use		7 (58)		6 (75)
HCV seropositive	1 (8)	1 (8)	0 (0)	0 (0)
Smoking	7 (58)	6 (50)	7 (88)	2 (25)
Cocaine use	6 (50)	6 (50)	4 (50)	4 (50)

Data shown are n (%) unless otherwise indicated

^α^ median [IQR]

### Characterization of plasma EV fractions

**Fig. 2:**
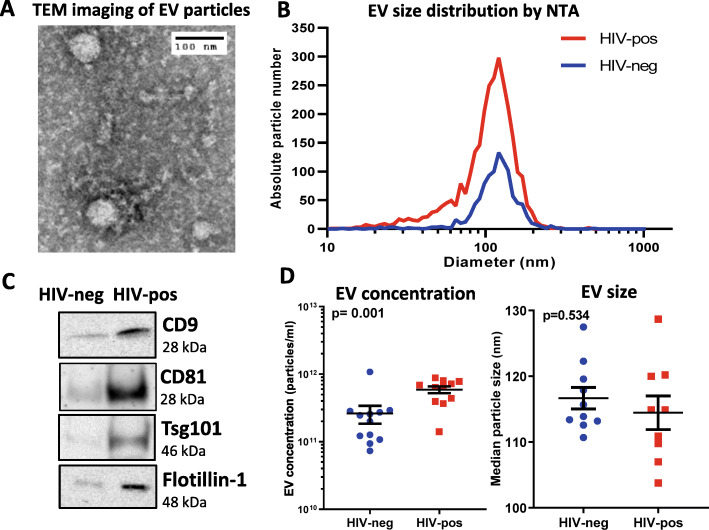
Characterization of EV fractions isolated from plasma. **a**. TEM image of isolated EV fraction from a HIV-negative control subject. **b**. Size distribution of EVs in a representative HIV-negative and HIV-positive subject by nanoparticle tracking analysis (NTA). **c**. Detection of exosome markers CD9, Flotillin-1, Tsg101, and CD81 by western blotting in pooled HIV-positive (*n* = 3) and HIV-negative (*n* = 3) samples. Three individual HIV-negative subjects were matched for age, gender, race, and cocaine use to three HIV-positive subjects to achieve matching between the pooled samples. Equal amounts of protein (50 μg per lane) were loaded for western blotting; these samples contain exosomal proteins, as well as co-purifying non-exosomal proteins from other sources such as microparticles and residual plasma proteins. Full-length blot images are shown in Supplementary Figure S6. **d**. EV concentration (left) and median EV size (right) analyzed by NTA in HIV-negative and HIV-positive subjects

EV fractions were isolated from HIV-positive (*n* = 12) and HIV-negative (n = 12) subjects in the test cohort using the PureExo exosome isolation kit and characterized by transmission electron microscopy (TEM), nanoparticle tracking analysis (NTA), and Western blotting for exosome markers (Fig. 2). TEM revealed vesicles of 40–70 nm in diameter (Fig. 2a), consistent with the size range of exosomes. NTA yielded distributions showing that majority of particles were 30–150 nm diameter, with a peak at 100–120 nm (Fig. 2b). Immunoblotting detected the exosome markers CD9, Flotillin-1, Tsg101, and CD81 in plasma EV fractions, with greater abundance of these markers detected in HIV-positive compared to HIV-negative subjects based on stronger band intensities (Fig. 2c). The endoplasmic reticulum (ER) marker calnexin was not detected by immunoblotting of these plasma EV fractions, suggesting they were free of ER membrane contamination and consistent with results of our previous study [12]. Based on NTA measurements, plasma EVs were more abundant in HIV-positive compared to HIV-negative subjects (mean 5.9 vs. 2.5 X 10^11^ particles/ml, respectively, in 30–150 nm size range; *p* = 0.001, Mann-Whitney test), while there was no significant difference in median particle size (114 vs. 116 nm, respectively) (Fig. 2d).

### Small RNA classes and distributions in plasma EVs of HIV-positive and HIV-negative subjects

**Fig. 3:**
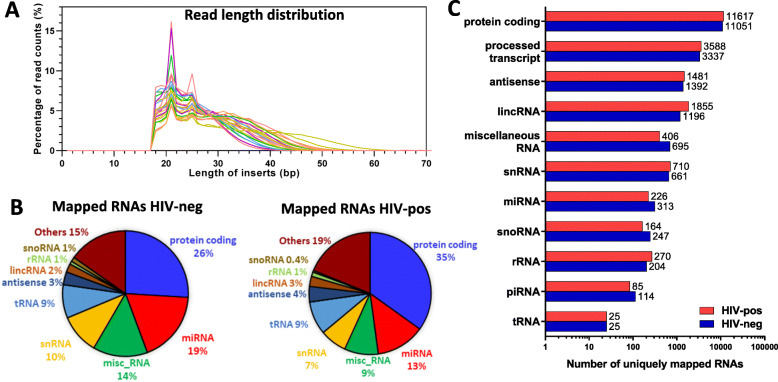
Mapping statistics of RNA species detected by small RNA-seq across 24 libraries. **a**. Size distribution of individual sequencing library inserts. **b**. Pie charts show distribution of small RNA species in plasma EVs of HIV-negative (left) and HIV-positive (right) subjects. Data shown are means of HIV-negative (*n* = 12) and HIV-positive (*n* = 12) subjects and depicted as percentage biotype counts. ‘Others’ category includes 36 additional RNA species listed in Supplementary Table 1. **c**. Total number of mapped unique RNA references in HIV-positive versus HIV-negative subjects

To characterize the plasma EV small RNA repertoire, we sequenced small RNA libraries from all subjects in the test cohort (12 HIV-positive and 12 HIV-negative) and mapped reads to the human genome (hg38) and small RNA databases (Fig. 1). Small RNA was isolated from EV fractions and quality was assessed using the BioAnalyzer platform with a small RNA chip, which showed that the quality of isolated small RNA was comparable between HIV-positive and HIV-negative groups. cDNA libraries were generated and subjected to single-read, 75-bp sequencing generating an average of 22 million reads per library. Raw read data was processed using the exceRpt Small RNA-seq Pipeline [22]. Reads were mapped to human rRNA to exclude rRNA sequences before mapping to the human genome. Size distributions among mapped reads in each sample showed that majority were between 16 nt to 60 nt in length with peaks at 21 nt and 26 nt, corresponding to miRNAs and piRNAs, respectively (Fig. 3a).

To examine the diversity of plasma EV RNA cargo, reads were mapped to human genome and small RNA databases for miRNA, piRNA, rRNA, tRNA, circular RNA, and snoRNA. Figure 3b shows a comparison of distributions of different RNA species identified in plasma EVs of HIV-negative (left) and HIV-positive (right) subjects in the test cohort. Protein coding reads were the most abundant RNA species in both groups, while miRNA was the most abundant small RNA species. A few subjects had miRNA as the most abundant RNA species instead of protein coding reads, or more snoRNA or tRNA as the most abundant small RNA species instead of miRNA; these findings were not associated with any distinctive subject characteristics. HIV-positive subjects had a higher proportion of protein coding reads compared to HIV-negative subjects (35% vs. 26%, respectively), and HIV-negative subjects had a higher proportion of miRNA reads compared to HIV-positive subjects (19% vs. 13%, respectively) (*p* < 0.0001, chi-square test). The next major classes of small RNAs detected were small nuclear RNA (snRNA), transfer RNA (tRNA), antisense RNA, long intergenic non-coding RNA (lincRNA), ribosomal RNA (rRNA), piRNA, small nucleolar RNA (snoRNA), and miscellaneous RNA (RNAs mapped to human genome, but not to any known RNA species in human genome). Supplementary Table 1 shows the complete list and total counts of each RNA species mapped across all samples. Number of uniquely mapped RNAs of different species is shown in Fig. 3c and summed read counts for each species are shown in Supplementary Figure S1. 26 and 35% of protein coding reads (Fig. 3b) mapped to 11,051 and 11,617 unique protein coding species in HIV-negative and HIV-positive subjects, respectively. Nineteen percent and 13% of miRNA reads mapped to 313 and 226 unique miRNAs in HIV-negative and HIV-positive subjects, respectively (Supplementary Table 2).

We identified a total of 14,500 unique protein coding sequences; among these, 392 (top 2.7%) had counts > 10 in at least 25% of the samples and accounted for 60% of all protein coding reads. Although fragments derived from protein coding sequences were the most abundant RNA species in terms of overall reads and number of different protein coding sequences, differential expression analysis of these protein coding sequences for the top 70 genes (filtered for genes with counts > 10 in at least 25% samples and not identified in blank samples) did not reveal any significantly altered protein coding sequences in HIV-positive vs. HIV-negative subjects after correction for multiple testing (Benjamini–Hochberg FDR adjusted *p*-value > 0.1) (Supplementary Table 3). The most abundant protein coding sequence reads were NPFFR1 (Neuropeptide FF Receptor 1), which is associated with G protein-coupled receptor activity and neuropeptide receptor activity, followed by WDR74 (WD Repeat Domain 74), a regulatory protein of the MTREX-exosome complex. Protein coding sequences for STEAP4 (STEAP4 Metalloreductase) and UTRN (Utrophin) showed an increasing trend in HIV-positive compared to HIV-negative subjects that did not reach significance (FDR > 0.1). Small nucleolar RNAs SNORD104, SNORD2, SNORD69, and SNORD63 were the most abundant snoRNAs, accounting for 50% of all snoRNA reads. Small nuclear RNAs U2, RNU1 and RNU2 were the predominant snRNAs, and were detected in majority of samples. PiRNA was the least abundant type of small RNA detected, with hsa-piR-018780 being the most abundant; however, majority of samples had counts < 10 for this piRNA. Mapping to the tRNA database identified 25 distinct tRNA species; among these tRNA^Gly^ (21%) and tRNA^Glu^ (20%) were the predominant tRNAs and were detected in all samples, while none of the remaining tRNAs exceeded 9% of total tRNA counts. These results show that circulating EVs in HIV-positive and HIV-negative subjects carry diverse small RNA species including miRNA, piRNA, snRNA, tRNA, and rRNA, with miRNAs being the most abundant small RNA species.

### Analysis of miRNA profiles in circulating EVs

**Fig. 4:**
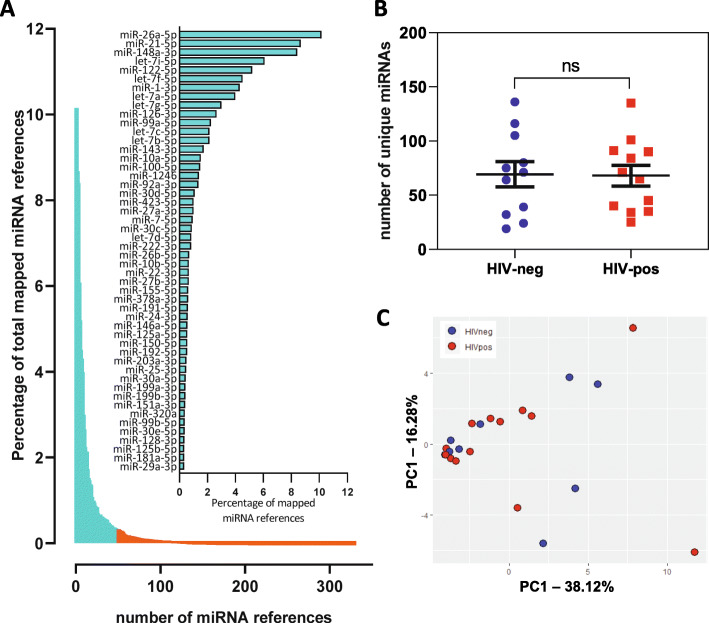
Small RNA-seq miRNA profiles in test cohort HIV-positive and HIV-negative subjects. **a**. Percentage (sorted from high to low) of each detected mature miRNA in all mapped miRNA reads in 24 subjects. The top 50 miRNAs (90%) are highlighted in aqua and shown in the embedded graph. **b**. Distribution of number of unique miRNAs in HIV-negative and HIV-positive subjects (*p* = 0.89, Mann-Whitney test). **c**. Principal component analysis of top 50 miRNAs in HIV-positive (red, *n* = 12) and HIV-negative subjects (blue, *n* = 8)

A total of 351 unique miRNAs were identified (Fig. 4a and Supplementary Table 2). The top 50 most abundant miRNAs accounted for 90% of all miRNA reads (aqua bars and embedded graph). miR-26a-5p was the most abundant miRNA, followed by miR-21-5p and miR-148-3p. HIV-positive and HIV-negative subjects had similar distributions of number of unique miRNAs detected (Fig. 4b, *p* = 0.89, Mann-Whitney test). To identify outliers, we performed principal component analysis (PCA) on the top 50 most abundant miRNAs. PCA analysis revealed 3 outliers (HIV-negative) (Supplementary Figure S2) that were excluded from downstream analyses, along with one sample with low number of identified miRNAs (0 count for > 94% of miRNAs). PCA analysis after outlier exclusion did not reveal distinct clusters differentiating HIV-positive and HIV-negative subjects (Fig. 4c). Additionally, there were no obvious outliers or clusters associated with age, race, cocaine, or smoking. Differential expression analysis of the top 50 miRNAs in HIV-positive vs. HIV-negative subjects is shown in Table 2. No miRNAs were significantly upregulated in HIV-positive compared to HIV-negative subjects by DEseq2 analysis after correction for multiple testing (FDR < 0.1), while only one miRNA (miR-181a-5p) was significantly downregulated (log2 FC = − 5.88, FDR-adjusted *p*-value = 0.0008). Two HCV-positive samples (HIVn-7 and HIVp-10) did not have distinctive miRNA profiles compared with HCV-negative samples in Supplementary Table 2, and analyzing the data after removing these two samples did not significantly alter the main findings in Table 2.

**Table 2:** Differential expression analysis of top 50 miRNAs in HIV-positive (*n* = 12) versus HIV-negative (*n* = 8) subjects from the test cohort

	baseMean	Log2 FC	*P* value	P adj
hsa-miR-26a-5p	620	−0.106	0.8216	0.9831
hsa-miR-148a-3p	514.38	0.798	0.2256	0.6636
hsa-miR-21-5p*	493.5	−0.405	0.4709	0.981
hsa-let-7i-5p	318.91	−0.191	0.7614	0.9831
hsa-miR-122-5p*	288.3	0.953	0.4941	0.9831
hsa-miR-1-3p	243.23	0.662	0.6196	0.9831
hsa-let-7a-5p*	224.95	1.512	0.0699	0.3493
hsa-let-7f-5p	216.84	−1.594	0.1263	0.4859
hsa-miR-423-5p*	210.36	0.239	0.8054	0.9831
hsa-let-7b-5p	181.67	1.098	0.2963	0.7134
hsa-miR-126-3p	134.04	3.067	**0.0056**	0.1169
hsa-let-7 g-5p	113.18	−0.108	0.9161	0.9831
hsa-let-7c-5p	109.04	2.39	**0.0466**	0.259
hsa-miR-30d-5p	102.88	−1.677	0.2039	0.6371
hsa-miR-99a-5p	96.3	0.527	0.6449	0.9831
hsa-miR-92a-3p	86.99	0.779	0.4643	0.981
hsa-miR-10a-5p*	83	2.9	**0.0213**	0.1779
hsa-miR-100-5p	80.05	1.389	0.2811	0.7134
hsa-miR-22-3p	79.59	2.307	0.0898	0.3743
hsa-miR-143-3p	73.61	1.308	0.3139	0.7134
hsa-miR-486-5p	69.46	1.373	0.3024	0.7134
hsa-miR-1246	64.35	−2.662	**0.0342**	0.2441
hsa-miR-155-5p	64.01	−0.322	0.7998	0.9831
hsa-miR-151a-3p	59.85	−0.261	0.8725	0.9831
hsa-miR-7-5p	57.03	0.038	0.9751	0.9917
hsa-miR-99b-5p	51.54	−0.933	0.7672	0.9831
hsa-miR-27a-3p	50.45	1.684	0.1728	0.5761
hsa-miR-320a	50.4	0.352	0.811	0.9831
hsa-miR-30c-5p	49.92	−1.558	0.2631	0.7134
hsa-let-7d-5p	44.98	2.287	**0.0391**	0.2447
hsa-miR-101-3p	44.39	−0.6	0.674	0.9831
hsa-miR-192-5p	41.22	2.429	0.0795	0.3615
hsa-miR-222-3p	41.18	0.194	0.8841	0.9831
hsa-miR-199a-3p	40.5	0.08	0.9438	0.9831
hsa-miR-199b-3p	40.5	0.08	0.9438	0.9831
hsa-miR-10b-5p	34.17	0.197	0.8896	0.9831
hsa-miR-181a-5p	33.66	−5.884	**1.59E-05**	0.0008
hsa-miR-27b-3p*	31.04	2.583	**0.0112**	0.1169
hsa-miR-26b-5p	30.29	0.012	0.9917	0.9917
hsa-miR-378a-3p	29.32	3.691	**0.0117**	0.1169
hsa-miR-24-3p	28.94	1.696	0.1589	0.5674
hsa-miR-191-5p	28.19	−0.71	0.676	0.9831
hsa-miR-25-3p	25.4	0.235	0.8851	0.9831
hsa-miR-125a-5p	22.86	−4.3	**0.0104**	0.1169
hsa-miR-451a	22.8	0.443	0.7961	0.9831
hsa-miR-146a-5p*	22.33	0.856	0.5596	0.9831
hsa-miR-125b-5p*	14.62	−0.665	0.6723	0.9831
hsa-miR-218-5p	14.38	1.004	0.5147	0.9831
hsa-miR-423-3p	12.6	0.485	0.7753	0.9831
hsa-miR-30a-5p	10.24	−0.284	0.8542	0.9831

* miRNAs selected for qRT-PCR validation are indicated with an asterisk. Significant raw *p*-values are in bold

### miRNAs associated with HIV infection, inflammation, and oxidative stress are increased in plasma EVs of HIV-positive compared with HIV-negative subjects

We selected 8 miRNAs identified by small RNA-sequencing (miR-27b-3p, − 21-5p, −125b-5p, − 122-5p, −10a-5p, − 423-5p, −146a-5p and let-7a-5p) for qRT-PCR validation based on the following criteria: an increasing trend in HIV-positive compared to HIV-negative by DEseq2 analysis (miR-10a-5p, − 122-5p, −146a-5p, −27b-3p, − 423-5p, and let-7a-5p), and/or previously shown to be associated with HIV infection (miR-122-5p, −125b-5p, −146a-5p, − 21-5p, −27b-3p, and − 423-5p) [23–27], or inflammation and oxidative stress (miR-10a-5p, −125b-5p, −146a-5p, − 21-5p, and -27b-3p) [28–31].

**Fig. 5:**
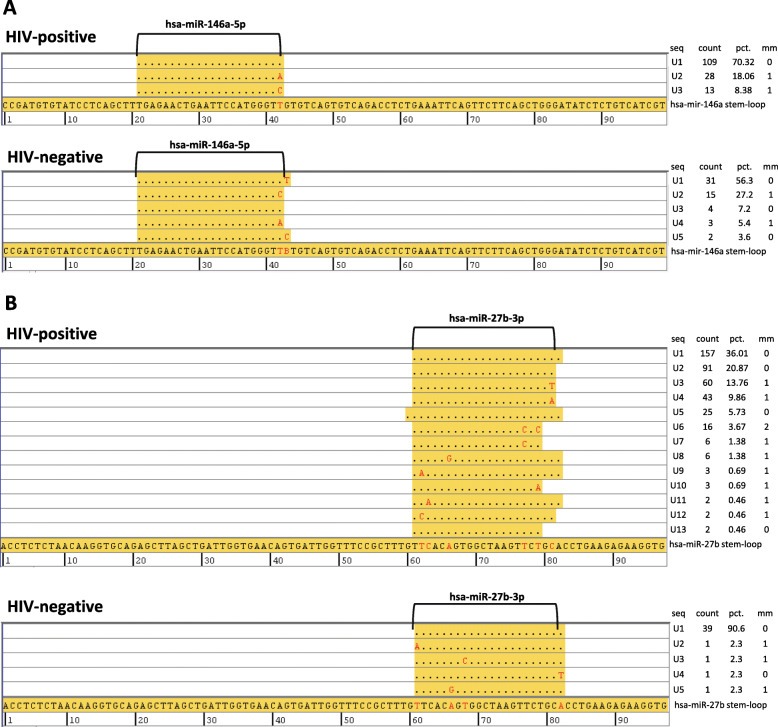
Alignment of miR-146a-5p and miR-27b-3p reads using CodonCode Aligner tool. miR-146a-5p (**a**) and miR-27b-3p (**b**) reads from representative HIV-positive (top) and HIV-negative samples (bottom) were aligned to corresponding miRNA stem-loop sequence from miRbase v21 showing matched nucleotides depicted by dots and mismatch nucleotides depicted by red font. Tables on the right show unique sequence number (seq), counts for each unique sequence (count), percentage of total counts (pct.), and number of mismatches (mm)

To confirm the identity of specific miRNAs detected by small RNA-seq, we performed manual alignment of miRNA reads using the CodonCode Aligner tool. To accomplish this, all reads from a sample mapping to a specific miRNA were manually aligned to the original miRNA stem-loop sequence from miRbase v21. Representative alignments for miR-27b-3p and miR-146a-5p from HIV-positive and negative samples are shown in Fig. 5, which shows that 100% of reads mapped to miR-146a-5p with 1 or 0 nucleotide mismatch and > 95% of reads mapped to miR-27b-3p with 1 or 0 nucleotide mismatch, suggesting these miRNAs and their isoforms (isomiRs) [32] are indeed present in these plasma EV samples.

The miRNAs in plasma are associated with lipoproteins (LDL/HDL) and ribonucleoproteins, which protect extracellular RNAs against RNase-mediated degradation and can be coprecipitated during EV isolation [33–35]. To exclude extra-vesicular miRNAs, EVs were isolated using the PureExo exosome isolation kit with the following modification: Proteinase K was added to plasma samples to release protein-associated miRNAs followed by RNase A treatment to degrade extravesicular RNAs. Proteinase K and RNase treatment resulted in reduction of small particles (10–30 nm) corresponding to lipoprotein particles as seen by TEM, particle size distribution, and particle concentration measurements (Supplementary Figure S3). Elimination of lipoprotein particles was further confirmed by Western blotting for ApoA1 (major structural protein component of HDL), soluble exosome markers (Alix and Tsg101), and membrane exosome markers (CD81- outer membrane and Flotillin-1 inner membrane). Proteinase K and RNase treatment degraded extra-exosomal proteins, while retaining intra-exosomal cargo as indicated by absence of CD81 and ApoA1 bands, and retention of Flotillin-1, Alix, and Tsg101 bands (Supplementary Figure S3).

**Fig. 6:**
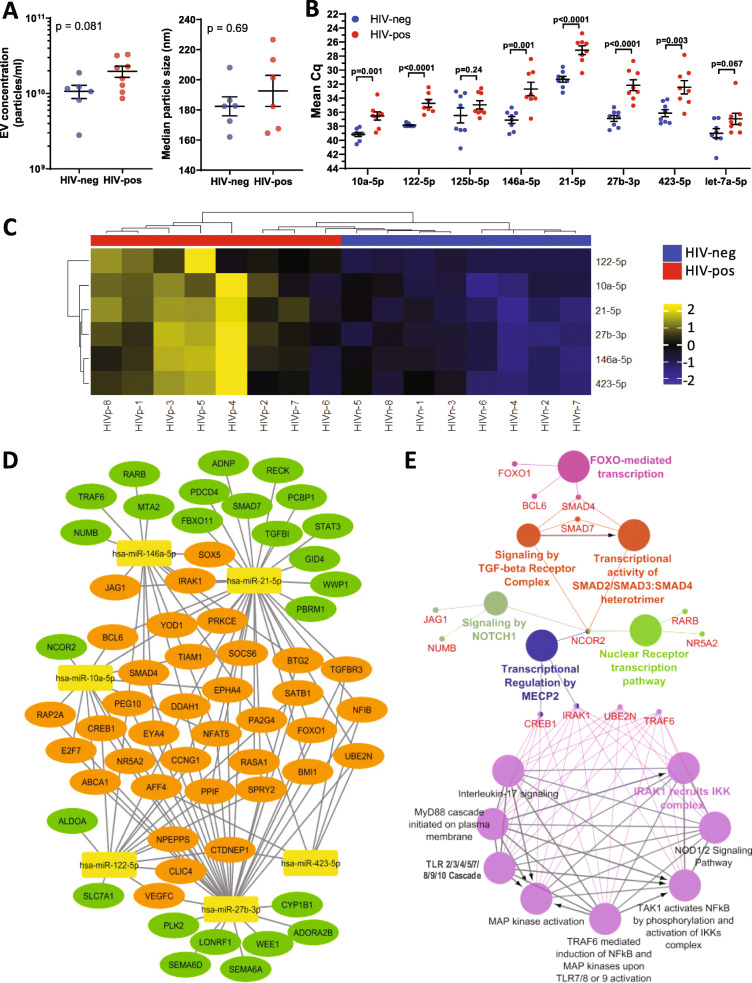
qPCR validation of candidate miRNAs and functional associations of differentially expressed miRNAs. **a** EV concentration (left) and median size (right) measured by NTA in exosome fractions isolated from HIV-positive and HIV-negative subjects from the validation cohort. **b** Comparison of Cq values of candidate miRNAs isolated from plasma EVs of HIV-positive and HIV-negative subjects using miRCURY LNA RT-qPCR kits. Mean and SEM are shown. Significance was calculated using Mann Whitney test. (*n* = 8 HIV-positive and *n* = 8 HIV-negative subjects). **c** Heatmap shows unsupervised hierarchical clustering of miRNAs (*n* = 6) that distinguish HIV-positive from HIV-negative subjects (*p* < 0.05, Mann-Whitney test). **d** . miRNA target–gene network was constructed using Cytoscape for 61 target genes of 6 differentially expressed miRNAs in the validation cohort. miRNAs are depicted by yellow nodes. 36 genes (60%) are targeted by more than one miRNA (orange nodes); genes targeted by one miRNA are depicted by green nodes. **e** Functionally grouped network of enriched categories was generated for miRNA-target genes with REACTOME pathways using the ClueGO plugin in Cytoscape as described in the Methods. Node sizes are indicative of *p*-values (larger size corresponds to smaller *p*-values and vice versa). Nodes belonging to one functional group share the same color. Similar pathway terms with identical genes were fused and pathway term ‘TP53 regulation of cell cycle genes’ was omitted for clarity. Complete list of pathway terms and associated genes is shown in Supplementary Table 6

To validate selected miRNAs in plasma EVs by qRT-PCR, we isolated small RNA from EV fractions of 8 HIV-positive and 8 HIV-negative subjects from the validation cohort. EV small RNA was isolated from equal plasma volumes and equal volumes of RNA were loaded for validation of candidate miRNAs by qRT-PCR analysis. Analysis of plasma EV fractions by NTA showed that HIV-positive subjects had higher EV numbers compared to HIV-negative subjects (Fig. 6a, *p* = 0.081), while EV size distribution was similar between groups. We detected significantly higher levels of miR-27b-3p, − 21-5p, − 122-5p, −10a-5p, −146a-5p, and − 423-5p in plasma EVs from HIV-positive compared to HIV-negative subjects by qRT-PCR (Fig. 6 b, *p* < 0.05, Mann-Whitney test), whereas miR-125b-5p and let-7a-5p did not show a significant difference (*p* = 0.24 and 0.067, respectively). As an additional control for qRT-PCR validation, we tested miR-7-5p, which showed no significant difference in plasma EVs from HIV-positive compared to HIV-negative subjects (*p* = 0.112), consistent with results in Table 2 (*p* = 0.991). Unsupervised hierarchical clustering in heatmaps show that significantly altered miRNAs (miR-27b-3p, − 21-5p, − 122-5p, −10a-5p, −146a-5p, and − 423-5p) distinguished between HIV-positive and HIV negative subjects based on z-scored qRT-PCR measurements (Fig. 6c). Given that cocaine abuse promotes oxidative stress and inflammation in HIV-positive subjects, we compared levels of these miRNAs between HIV-positive and HIV-negative subjects stratified by cocaine use. These miRNAs remained elevated in HIV-positive cocaine users and non-users compared to HIV-negative cocaine users and non-users, respectively (Supplementary Figure S4). In contrast, there was no change in miRNA levels associated with cocaine use within the HIV-positive or HIV-negative groups. Thus, the observed changes in plasma EV miRNA levels were likely related to effects of HIV infection rather than cocaine use.

### Functional over-representation analysis of miRNAs

We performed functional over-representation analysis (ORA) of differentially expressed miRNAs identified in the validation cohort using the web-based application miEAA (miRNA Enrichment Analysis and Annotation). ORA of miRNAs: miR-27b-3p, − 21-5p, − 122-5p, −10a-5p, −146a-5p, and − 423-5p found significant enrichment of categories: Pathways (176 terms), Gene Ontology (GO, 732 terms), and Diseases (7 terms). ORA results are shown in Supplementary Table 4; HIV-positive versus HIV-negative subjects had significant enrichment of miRNA targets mapping to pathways such as oxidative stress response, interferon gamma signaling, Toll-like receptor signaling, T cell activation, TGF beta signaling, and Notch signaling among the top 20 most significant pathways (*p* = 0.008). Among the top 25 GO terms enriched were response to stress, positive regulation of T cell cytokine production, cytoplasmic membrane bound vesicle, microtubule-based movement, late endosome, and endocytosis (*p* ≤ 0.008). Furthermore, ≥ 5 of 6 up-regulated miRNAs supported these ORA results (‘observed’ column in Supplementary Table 4). Disease terms enriched by ORA were autoimmune diseases, inflammation, metabolic diseases, and obesity (*p* < 0.05). These results suggest that miRNAs associated with factors related to HIV infection and EV secretion are enriched in plasma EVs of HIV-positive subjects.

### miRNA-target enrichment and functional analysis

Target genes of differentially expressed miRNAs identified in the validation cohort (miR-27b-3p, − 21-5p, − 122-5p, −10a-5p, −146a-5p, and − 423-5p) were predicted using predictor algorithms queried by miRDIP v4.1.11.1 and miRWalk v3.0, along with experimentally validated targets deposited in miRTarbase v7.0. The complex interaction relationship between these 6 miRNAs and their target genes was visualized as a network (Fig. 6d) for a set of 61 predicted and validated targets (Supplementary Table 5). miR-21-5p and miR-27b-3p showed the highest number of overall target genes (*n* = 33 genes each), followed by miR146a-5p and miR-122-5p. Majority of these target genes were associated with more than one miRNA (36 genes, orange nodes). For example, NFAT5 is a predicted target of 5 of the 6 miRNAs, while DDHA1 is a predicted target of 4 of the 6 miRNAs. We performed functional enrichment analysis of the miRNA-target genes with REACTOME pathways using the ClueGO v2.5.5 plugin of Cytoscape 3.7.2 (Fig. 6e). A set of 31 statistically significant pathways (*p* < 0.005) were identified including Toll-like receptor signaling cascade, MyD88 cascade, IRAK1 recruits IKK complex, TGF-beta receptor signaling, Notch signaling pathway, and NOD1/2 signaling (Supplementary Table 6), in accordance with the over-representation analysis (Supplementary Table 4). TNF receptor-associated factor 6 (TRAF6) and IL-1 receptor associated kinase (IRAK1) genes were associated with majority of these pathway terms; both genes are predicted and validated targets of miR-146a-5p.

### EV miRNAs correlate with oxidative stress markers

**Fig. 7:**
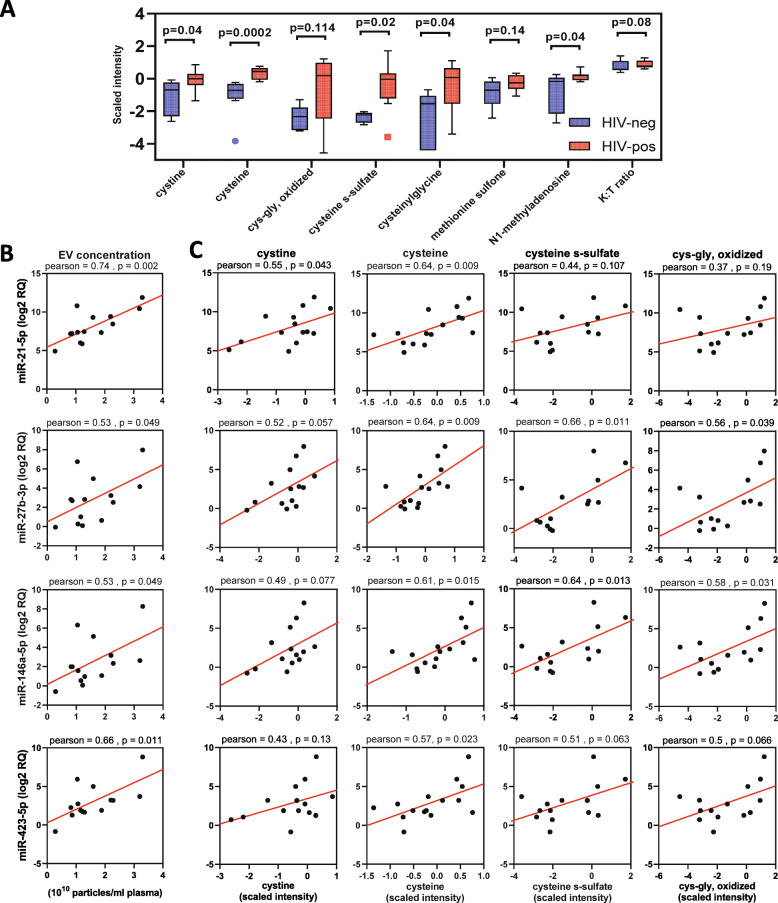
Correlation of candidate miRNAs with EV numbers and oxidative stress associated metabolites. **a**. Metabolites associated with oxidative stress and immune activation are increased in HIV-positive versus HIV-negative subjects. Medians are represented by horizontal bars, boxes span the IQR, and whiskers extend to extreme data points within 1.5 times IQR. Outliers are plotted outside 1.5 times the IQR. *P*-values were calculated by Mann-Whitney test (*n* = 8 HIV-negative, *n* = 8 HIV-positive subjects). Scatter plots show correlation of miR-21-5p, miR-27b-3p, miR-146a-5p, and miR-423-5p with EV numbers (**b**) and oxidative stress associated metabolites cystine, cysteine, cysteine-s-sulfate, and cys-gly, oxidized (**c**) in HIV-positive (*n* = 8) and HIV-negative (*n* = 8) subjects. Correlation coefficients and *p*-values are shown above each plot. K:T ratio, kynurenine:tryptophan ratio; RQ, relative quantity

We previously showed positive correlation of oxidative stress metabolites (methionine and cysteine metabolism) and kynurenine:tryptophan ratio (K:T ratio, immune activation marker), and negative correlation of anti-inflammatory polyunsaturated fatty acid (PUFA) metabolites (n-3 and n-6 PUFA metabolism) with exosome markers [12]. Based on these findings, we performed untargeted metabolomic profiling of plasma from subjects in the validation cohort using the platform described in [12], which detected 655 endogenous metabolites from which we selected 12 metabolites or ratios (Supplementary Table 7) related to: 1) tryptophan catabolism (increased with immune activation) [36, 37]; 2) methionine and cysteine metabolism (altered with oxidative stress) [38, 39]; and 3) n-3 and n-6 PUFA metabolism (anti-inflammatory pathway). Metabolite markers of oxidative stress (cysteine, cystine, cysteine s-sulfate, cysteinyl glycine, N1-methyladenosine; *p* < 0.05, Mann-Whitney test) and K:T ratio (*p* = 0.08) were increased in HIV-positive compared to HIV-negative subjects; two additional markers of oxidative stress (cysteinyl glycine, oxidized and methionine sulfone) showed similar trends (Fig. 7a). Given greater abundance of miR-27b-3p, − 21-5p, −146a-5p, and − 423-5p in HIV-positive vs. control subjects in the validation cohort, we examined relationships between these miRNAs and plasma metabolite markers of oxidative stress, immune activation, and EV abundance in HIV-positive and HIV-negative subjects. Increased EV numbers in HIV-positive subjects correlated positively with miR-27b-3p, − 21-5p, −146a-5p, and − 423-5p (Fig. 7 b, *p* < 0.05). Metabolites of the cysteine metabolism pathway (i.e. cysteine, cystine, oxidized cys-gly, cysteine s-sulfate) correlated positively (Fig. 7c), while PUFA metabolites (docosahexaenoate (22:6n3) (DHA), n-3 (22:5n3) and n-6 (22:5n6) docosapentaenoate (DPA), and eicosapentaenoate (20:5n3) (EPA)) correlated negatively with each of the 4 miRNAs tested (Supplementary Figure S5). The immune activation marker K:T ratio did not correlate with any of the miRNAs tested (data not shown). We also tested correlation of miRNAs miR-10a-5p and miR-122-5p with these metabolites, but did not find any significant correlations (data not shown).

## Discussion

In this study, we applied next generation small RNA sequencing to characterize RNA cargo of plasma EVs in HIV-positive subjects on ART relative to healthy controls and evaluated abundance of different RNA species and association of miRNAs with disease-related factors. To our knowledge, this is the first study exploring EV small RNA cargo in treated HIV patients. We found higher abundance of plasma EVs in treated HIV-positive subjects compared to HIV-negative controls, consistent with our previous study [12]. Small RNA sequencing of libraries from plasma EVs showed similar sequencing depth between HIV-positive and HIV-negative subjects (23 versus 21.8 million average reads, respectively). Mapping to the human genome and small RNA databases revealed distributions of diverse coding and non-coding RNA species including protein coding sequences, miRNA, rRNA, tRNA, lincRNA, snRNA, and snoRNA, with protein coding sequences being the most abundant RNA species and miRNA being the most abundant small RNA. Consistent with our findings, a previous study of plasma EVs from healthy volunteers identified protein coding sequences as the most abundant RNA species [40]. Yuan T et al. reported miRNAs as the most abundant plasma extracellular RNA species followed by piRNAs in a study of cancer patients [41], whereas piRNA was among the least abundant RNA species in our study. Based on detection of mostly exosome-sized vesicles in isolated EV fractions together with prior studies showing miRNAs enrichment in exosomes, EV miRNAs characterized in our study most likely reflect exosomal miRNAs.

miRNAs are abundant in biofluids and widely studied as biomarkers of diseases and biological processes. We detected a total of 351 different miRNAs in plasma EVs, with the top 50 miRNAs accounting for 90% of all miRNA reads. miR-26a-5p was the most abundant miRNA, followed by miR-21-5p and miR-148-3p, consistent with another report studying small RNA signatures in different body fluids where miR-26a-5p and miR-21-5p were the most abundant miRNAs in plasma [42]. Liver specific miRNAs miR-122 and miR-22 were among the top 50 miRNAs identified in the test cohort, suggesting liver is one potential source. miR-122 showed an increasing trend in the test cohort and was significantly elevated in HIV-positive subjects in the validation cohort. Elevated miR-122 is implicated in viral hepatitis and contributes to hepatotoxicity in HIV-positive individuals on ART [20, 43, 44]. Other abundant sources of miRNAs in plasma are LDL and HDL particles. While LDL-miRNA profiles align more closely with EV-miRNAs, HDL-miRNAs have distinct miRNA profiles [35]. Importantly, miR-135a-3p, an HDL-associated miRNA, was not among the top 50 miRNAs detected in our samples. Likewise, a platelet-associated miRNA, miR-223, was not detected in majority of samples (mean count < 5), suggesting platelets were not a major source of detected miRNAs. The diversity of miRNA species was similar in HIV-positive and HIV-negative subjects (Fig. 4b), and we did not detect any significantly altered miRNAs in HIV-positive versus HIV-negative subjects by DEseq2-differential expression analysis of the top 50 miRNAs. The inability of DEseq2 to detect significantly altered miRNAs in our study could be due to the low RNA input from small volume of plasma used to isolate EV fractions, and/or PCR bias during library preparation.

We selected miRNAs showing an increasing trend in HIV-positive subjects in the test cohort, or previously linked to HIV infection, inflammation, and/or oxidative stress, for qRT-PCR validation in an independent cohort, and confirmed the identity of selected miRNAs by aligning reads to their corresponding stem-loop sequence from miRbase. Six miRNAs quantified by qRT-PCR (miR-27b-3p, − 21-5p, − 122-5p, −10a-5p, − 423-5p, and -146a-5p) were increased in HIV-positive compared to HIV-negative subjects in the validation cohort. Possible reasons why these six miRNAs were significantly increased in the validation cohort, while only two of these (miR-27b-3p and -10a-5p) had a statistically significant increase (unadjusted *p* < 0.05) in the test cohort, include the following: 1) validation cohort consisted of HIV-positive subjects with more advanced HIV disease compared to the test cohort; 2) higher specificity of targeted qRT-PCR as compared to whole library amplification during RNA-seq; and 3) normalization was performed for the test cohort during differential expression analysis by DEseq2, while Cq values from qRT-PCR were directly compared in the validation cohort for equal volumes of starting plasma and EV RNA.

Among the six miRNAs elevated in plasma EVs of HIV-positive subjects in the validation cohort, miR-125b-5p, −146a-5p, − 21-5p, −27b-3p, and − 423-5p were previously associated with HIV infection. miR-125b-5p may play a role in HIV latency [25], while miR-146a is elevated in plasma of HIV-positive subjects, and may inhibit HIV by disrupting RNA-mediated Gag assembly and virion budding [24, 45]. Elevated levels of miR-27b decreased viral gene expression levels of HIV in vitro [23]. Thus, increased levels of these miRNAs may play a protective role during HIV infection. miR-27b and other miRNAs including miR-10a, − 21, −125b, and -146a are increased in vascular tissues during inflammation and oxidative stress [28]. One factor that can promote inflammation and oxidative stress in HIV-positive subjects is cocaine abuse [46, 47], which was prevalent in both the test and validation cohorts (50%). However, we did not detect significantly altered miRNAs with cocaine use in HIV-positive or healthy control groups in either cohort.

Our previous studies identified changes in plasma metabolites related to chronic immune activation, oxidative stress, and inflammation in HIV patients on ART [12]. Oxidative stress metabolites correlated positively, while anti-inflammatory PUFA metabolites correlated negatively, with exosome markers. Oxidative stress increases exosome secretion in vitro, which can communicate protective messages to other cells in part by exosomal shuttling of RNA cargo [48]. EV-associated miRNAs, miR-27b-3p, − 21-5p, −146a-5p, and − 423-5p were increased in HIV-positive subjects compared to HIV-negative controls and correlated positively with metabolites associated with oxidative stress (cysteine, cystine, oxidized cys-gly, cysteine s-sulfate). miRNAs miR-27b and miR-21 are increased in response to oxidative stress in macrophages and regulate macrophage functions via the NF-kB pathway [49], while miR-423 targets the HIV genome in Gag regions and may interfere with HIV replication [50]. Our finding that oxidative stress- and HIV-associated miRNAs within plasma EVs correlate positively with oxidative stress metabolites is consistent with a functional role for exosomes in modulating HIV pathogenesis and redox homeostasis.

Over-representation and pathway enrichment analysis of differentially expressed miRNAs and their target genes predicted functional associations of these upregulated EV miRNAs with oxidative stress response, interferon gamma signaling, Toll-like receptor signaling, TGF beta signaling, and Notch signaling. Interestingly, our previous report investigating the EV proteome identified Notch4 in plasma EVs and showed that EV-associated Notch4 was increased in HIV-positive individuals and correlated with immune activation markers [12]. Majority of terms in pathway enrichment analysis were associated with TRAF6 and IRAK1 genes, which are targeted by miR-146a-5p [51]. miR-146a-5p negatively regulates type 1 interferon and inflammatory cytokine production by targeting TRAF6 and IRAK1 [52, 53]. Transfection of a miR-146a mimic in THP-1 cells leads to reduction in levels of major cytokines/chemokines induced by LPS [52]. Collectively, these data suggest that increased levels of these miRNAs in circulating EVs may have protective anti-inflammatory effects during HIV pathogenesis.

We acknowledge limitations of the study, particularly those related to purity of EV preparations [54]. Although plasma is a good source of EVs, it is challenging to separate plasma EVs from abundant plasma proteins, larger microvesicles, lipoprotein particles, and ribonucleoproteins. In plasma, RNAs are associated with lipoproteins (LDL/HDL) and ribonucleoproteins, which protect extracellular RNAs against RNase-mediated degradation; these particles can be coprecipitated during EV isolation, leading to contamination of EV preparations with extravesicular RNAs. In the validation cohort, we attempted to eliminate extravesicular RNAs by Proteinase K followed by RNase A treatment. This method resulted in higher purity of EV fractions, but lower EV yield. Our study was also limited by small volumes of plasma available for EV RNA isolation, leading to low RNA input. Batch effects could be another confounding factor. Additional potential confounders include effects of smoking, HCV infection, and ART treatment, which could influence some findings [55, 56]. In particular, given known effects of smoking on inflammation and miRNA expression and different proportions of smokers between groups in the validation cohort, we cannot exclude the possibility that smoking contributed to some of our results. Further studies in larger cohorts are needed to address the impact of cigarette smoking, HCV infection, ART drugs, and other factors on EV RNA cargo and to assess whether target genes of upregulated miRNAs identified in plasma EVs are downregulated in cells from the same individuals.

## Conclusions

In conclusion, our study shows that HIV-positive individuals on ART have elevated abundance of circulating EVs compared to HIV-negative individuals, and these EVs carry diverse small RNA cargo including miRNA, piRNA, snRNA, tRNA, and snoRNA, with miRNAs being the most abundant. Given their functional role in post-transcriptional regulation, we focused on miRNAs for validation studies and showed that six miRNAs (miR-27b-3p, − 21-5p, − 122-5p, −10a-5p, − 423-5p, and -146a-5p) were increased in plasma EVs of HIV-positive compared to HIV-negative individuals, and these miRNAs correlated with metabolite markers of inflammation and oxidative stress. These upregulated EV miRNAs are predicted to have functional associations with oxidative stress responses, interferon gamma signaling, Toll-like receptor signaling, TGF beta signaling, and Notch signaling based on over-representation and pathway enrichment analyses. These findings suggest that circulating EV miRNAs may reflect ongoing pathophysiological processes in HIV-infected individuals on ART, serving as potential biomarkers of inflammation and oxidative stress and targetable mechanisms involved in disease pathogenesis.

## Methods

### Study subjects

The study was performed in accordance with guidelines in the Declaration of Helsinki. Test cohort plasma samples were obtained from HIV-positive (*n* = 12, age 43–60 years) and HIV-negative subjects (n = 12, age 31–62 years) enrolled in the Chicago site of the Multicenter AIDS Cohort Study (MACS), an ongoing prospective study of HIV-infected and -uninfected MSM. All subjects were enrolled with written informed consent and IRB approval at Northwestern University Feinberg School of Medicine. Validation cohort plasma samples were obtained from HIV-positive subjects (*n* = 8, age 45–67 years) enrolled in the National NeuroAIDS Tissue Consortium (NNTC) and healthy control plasma samples (n = 8), were from HIV-negative donors (from Bioreclamation IVT) with informed consent and IRB approval at each NNTC study site (Manhattan HIV Brain Bank, National Neurological AIDS Bank, California NeuroAIDS Tissue Network, Texas NeuroAIDS Research Center) and Dana-Farber Cancer Institute, respectively. Subjects were tested for HCV and HBV, but not HIV-2, HTLV-I/II, or other infectious agents. HIV-negative groups were frequency-matched to corresponding HIV-positive groups to achieve overall balance in distributions of age, race, cocaine use, and HCV infection between the HIV-negative and HIV-positive groups in test and validation cohorts. Inclusion criteria for HIV-positive subjects in both cohorts were: adults over 40 years old on ART with HIV plasma viral load undetectable or below 1000 HIV RNA copies/ml. Exclusion criteria for all subjects were testing positive for HBV surface antigen and/or HBV DNA.

### EV isolation and size measurements

Fresh frozen plasma samples (0.5 ml) were thawed and centrifuged for 15 min at 3000 X g. Cleared plasma was incubated with thromboplastin D for 5 min at room temperature to de-fibrinate plasma, followed by centrifugation at 10,600 x g for 5 min. RNAse A treatment was performed (10 μg/ml, at 37 °C for 15 min) to degrade extra-exosomal RNA, followed by addition of RNAse inhibitor (150 U/ml, New England Biolabs) [57]. EV fractions were isolated using PureExo Exosome Isolation kit (101Bio, Mountain View, CA) per manufacturer’s instructions. Treated plasma was mixed with sample buffer and a 1:1:1 mixture of reagent A, B, and C was added and mixed by inverting tubes several times. The mixture was incubated at 4 °C for 1 h and centrifuged at 5000 X g for 3 min. The middle fluffy layer containing EVs was collected and final EV pellet was resuspended in PBS. The resulting EV fraction was passed through 0.22 μm filter to remove EVs larger than exosomes. EV morphology was characterized by imaging EV fractions on a Tecnai G2 Spirit BioTWIN Transmission Electron Microscope (TEM) equipped with an AMT 2 k CCD camera at the Harvard University TEM core. Size distribution and concentration of EV fractions was measured by nanoparticle tracking analysis (NTA) on a ZetaView instrument (Particle Metrix).

### Small RNA isolation, library construction, and high-throughput sequencing

Seven hundred μl Qiazol was added to EV fractions and frozen at − 80 °C. Small RNA was isolated using miRNeasy micro kit (Qiagen) as per manufacturer’s instructions. RNA quality was assessed with Agilent BioAnalyzer using a small RNA chip. All samples were treated with T4 polynucleotide kinase (New England Biolabs) to facilitate 5′ hydroxyl terminus phosphate labelling and allow greater binding of adaptors during library preparation [58]. Small RNA libraries were prepared by processing 12 samples per batch (6 HIV-positive and 6 HIV-negative), using NEBNext small RNA Library Prep kit (New England BioLabs) according to the manufacturer’s instructions, except 5′ and 3′ adaptors were diluted 1:3 with nuclease-free water to reduce adaptor dimer formation. The amplified libraries were resolved on a 10% Novex TBE gel (Life technologies) and a library size range from 140 to 160 bp (derived from adapter-ligated constructs from 21 to 40 nucleotide RNA fragments) was excised from the gel for size selection and recovered in DNA elution buffer. Average size distribution of each library was determined with the Agilent Bioanalyzer System using High Sensitivity DNA Analysis Kit and quantified on ABI 7900HT Fast RT-PCR instrument using the KAPA Library Quantification kit. All libraries were pooled and sequenced on the Illumina NextSeq 500 platform for single read 75 cycles at the Center for Cancer Computational Biology, Dana-Farber Cancer Institute.

### Small RNA sequence data processing and mapping

Raw sequence data from Illumina NextSeq 500 were converted to fastq format. Small-RNAseq reads were processed and quantified using the exceRpt small RNA-seq pipeline (version 4.6.2) available on the Genboree Workbench [http://www.genboree.org/] (Fig. 1). The software processes each sample independently through a cascade of read-alignment and filtering steps designed to remove likely contaminants before aligning to endogenous sequence databases. Adapters were trimmed and read quality assessed by FASTQC to filter out reads with a PHRED score lower than 30 (FASTX-Toolkit v0.0.13). Reads < 16 nt were excluded. Likely contaminant sequences derived from laboratory or rRNA contamination were removed by mapping to the UniVec (library of common contaminant sequences maintained by the NCBI) and human ribosomal RNA (rRNA) sequences using Bowtie2. Post filtering, reads were mapped to human genome and pre-miRNA sequence databases allowing for only a single mismatched base in each alignment. First, reads were mapped to miRbase version 21, gtRNAdb, piRNABank, circBase, and snoRNA-LBME databases to assign reads to miRNAs, tRNAs, piRNAs, circular RNAs, and snoRNAs, respectively. Then, remaining sequences were annotated to gencode version 24 (hg38), which includes biotypes such as protein coding transcripts, mitochondrial rRNA, mitochondrial tRNA, small nuclear RNA, long intergenic noncoding RNA (lincRNA), pseudogenes, and miscellaneous RNA.

### qPCR validation of miRNAs

To validate candidate miRNAs from sequencing data, we performed qPCR analysis of miR-10a-5p, miR-122-5p, miR-125b-5p, miR-146a-5p, miR-21-5p, miR-27b-3p, miR-423-5p, and let-7a-5p. miRNA-specific miScript Primer Assays were purchased from QIAGEN (hsa-miR-10a-5p: YP00204778, hsa-miR-122-5p: YP00205664, hsa-miR-125b-5p: YP00205713, hsa-miR-146a-5p: YP00204688, hsa-miR-21-5p: YP00204230, hsa-miR-27b-3p: YP00205915, hsa-miR-423-5p: YP00205624, hsa-let-7a-5p: YP00205727, and spike-in control, UniSp6: YP00203954). EV fractions were isolated following the same protocol as described under ‘EV isolation and size measurements’ with the following modifications to eliminate extravesicular RNAs: defibrinated plasma was treated with proteinase-K (0.5 mg/ml for 30 min at 55 °C) to release protein-associated RNAs [34, 59]. Following isolation of EV fraction, PMSF (5 mM final concentration) was added to inhibit proteases. RNAse A and RNAse inhibitor treatment was performed on EV fractions as described in ‘EV isolation and size measurements’. UniSp6 spike-in RNA was added to each sample lysate and RNA isolation was performed as described above and eluted in 14 μl nuclease-free water. Equal volumes of EV RNA (6 μl) from each sample were reverse transcribed using the miRCURY LNA RT Kit (Qiagen) at 42 °C for 60 min, and then the enzyme was inactivated at 95 °C for 5 min. After activation of the polymerase enzyme at 95 °C for 2 min, 45 cycles of 95 °C for 10 s, and 56 °C for 60 s were performed using miRCURY LNA SYBR Green PCR Kit (Qiagen), on the BioRad CFX96 Real-Time System. Since qPCR was performed in multiple batches, UniSp6 spike-in was assayed in each batch to monitor variation between batches. Each miRNA was assayed in duplicate and means of 2 Cq values were calculated. Cq values were normalized (ΔCq) by the global mean to account for inter-individual sample differences.

### Western blot analysis

EV fractions were lysed in lysis buffer (Triton X-100 1%, NaCl 150 mM, sodium deoxycholate 0.5%, Tris-HCL 50 mM, SDS 0.1%, pH 7.4) and protein content measured by BioRad DC protein assay. Fifty micrograms of protein were separated in each lane of Tris SDS polyacrylamide gels (4–12% gradient) and transferred onto PVDF membranes. Blots were blocked with 5% milk and probed overnight at 4 °C with primary antibodies against exosome markers CD9, Alix (sc-59,140, sc-53,540, SantaCruz Biotechnology), CD81 (NB100–65805, NovusBio), Flotillin-1, Tsg101 (610,821, 612,696, BD BioScience), and Apo A1 (3710–3-1000, MabTech), followed by appropriate secondary antibodies for 1 h; signal was developed by enhanced chemiluminescence (ECL). Images were captured using a BioRad ChemiDoc™ Imaging System.

### Metabolomic profiling

Untargeted metabolomic profiling was performed by Metabolon (Durham, NC) combining three independent platforms: ultra-high performance liquid chromatography and tandem mass spectrometry (UHLC/MS2/MS) optimized for detection of acidic metabolites, UHLC/MS2/MS optimized for detection of basic metabolites, and gas chromatography (GC)/MS. Plasma samples (100 μl) were extracted using the MicroLab STAR system and processed for analysis on the three platforms as described [12]. Samples derived from pooled experimental samples served as technical replicates, extracted water samples served as blanks, and a cocktail of standards spiked into every analyzed sample allowed instrument performance monitoring. Compounds were identified by automated comparison of the ion features in the experimental samples to a reference library of over 4000 chemical standard entries that included retention time, molecular weight (m/z), preferred adducts, and in-source fragments as well as associated MS spectra and curated by visual inspection for quality control using software developed at Metabolon.

### Functional over-representation analysis of miRNAs

We assessed functions of differentially expressed miRNAs from HIV-positive versus HIV-negative subjects using miRNA enrichment analysis and annotation tool (miEAA; https://ccb-compute2.cs.uni-saarland.de/mieaa_tool/ accessed in December 2019) [60]. MiEAA is a web-based application that offers a variety of commonly applied statistical tests such as over-representation analysis (ORA) and facilitates the functional analysis of sets of miRNAs. MiEAA performs rich functional analysis in terms of miRNA categories such as gene ontology, pathways, diseases, immune cells, and species conservation, and tests whether a category is significantly enriched (FDR adjustment) in a given miRNA set with respect to a reference using statistical tests implemented in the gene set analysis toolkit, GeneTrail.

### Identification of miRNA-target genes

Predicted and validated miRNA–target relationships were assessed by the web-based multiple predictor tools mirDIP v4.1.11.1 [61], and miRWalK v3.0 [62]. These tools query a series of miRNA target predictors and show which target is predicted by one or more algorithms. We also used experimentally validated targets deposited in miRTarBase v7.0 [63]. Targets from mirDIP were filtered to include only those that were predicted by at least 20 different sources and score class was set to ‘very high’ (top 1%). Targets from miRWalk were filtered to include target genes present in all three databases queried by miRWalk (TargetScan 7.2, miRDB 5.0, and miRTarBase 7.0). The final set of target genes (Supplementary Table 5) for validated miRNAs was selected based on: 1) being present in both miRDIP and miRWalk filtered lists; or 2) present in either list and experimentally validated in miRTarbase. Predicted miRNA target–gene networks were constructed using Cytoscape v3.7.2 [64].

### Pathway enrichment analysis

Functional enrichment analysis of miRNA-target genes was performed with REACTOME pathways using the ClueGO v2.5.5 [65] plugin of Cytoscape 3.7.2. To identify enriched pathways in the REACTOME database, we used two-sided (enrichment/depletion) tests based on hyper-geometric distribution. Pathways with *p* ≤ 0.05 were selected and Benjamini-Hochberg adjustment was used to correct *p*-values for terms and groups created by ClueGO. Kappa score threshold was set to 0.6; GO tree interval was 3–8; Leading Group was selected by ‘highest significance’; % of Group Merge was 50.

### Data processing and statistical analysis

For small RNA-sequence data analysis, raw read counts obtained from the Genboree Workbench’s exceRpt small RNA-seq pipeline were further analyzed using R (version 3.5.2). Differential expression fold-changes and *p*-values were calculated with the DEseq2 package (version 1.22.2), adjusting for library concentration, in conjunction with adjustment for ‘nuisance factors’ estimated from glm residuals using the RUVseq package (version 1.16.1). Altered miRNAs or protein coding sequences with absolute fold change > 1.3 and FDR < 0.1 were considered significant. miRNAs with < 5 counts per million reads (cpm) in ≥75% of the samples were excluded and the top 50 most abundant miRNAs were selected for DE analysis. Unsupervised hierarchical clustering using the ComplexHeatmap R package (version 2.2.0) was used to evaluate clustering in heatmaps. For metabolite profiling, metabolite data was normalized by median centering. Missing values were imputed with the lower limit of detection for a given metabolite. Pearson correlations were used to evaluate relationships between plasma metabolites and miRNA levels (*p* < 0.05). qPCR miRNA levels were compared between groups using the Mann Whitney U-test in PRISM (GraphPad) (*p* < 0.05).

## Supplementary Information

**Additional file 1: Supplementary Figure S1:** Comparison of total read counts for each RNA biotype in HIV-positive (*n* = 12) versus HIV-negative (*n* = 12) subjects. **Supplementary Figure S2**. Principal component analysis of the top 50 miRNAs identified by small RNA sequencing of plasma EV RNAs from HIV-positive (*n* = 12) and HIV-negative (*n* = 12) subjects. Three HIV-negative outliers (circled in green) were excluded from downstream differential expression analysis. **Supplementary Figure S3**. Plasma EV isolation and purification to exclude extravesicular RNAs. EV fractions were isolated from pooled plasma of healthy control subjects (*n* = 3) using the PureExo kit. Defibrinated plasma was either untreated, or treated with RNAse A, or with Proteinase-K followed by RNAse A, to eliminate extravesicular RNAs. TEM (top), particle size distribution (bottom left), particle concentration (bottom middle), and immunoblotting for exosome markers and ApoA1 (bottom right) are shown for each treatment condition. **Supplementary Figure S4**. Comparison of Cq values of miRNAs in plasma EVs of HIV-positive and HIV-negative subjects in the validation cohort, stratified by cocaine use. Mean and SEM are shown. Significance was calculated using Mann Whitney test. (*n* = 8 HIV-positive and *n* = 8 HIV-negative subjects). **Supplementary Figure S5:** Scatter plots showing inverse relationships between PUFA metabolites and EV-associated miRNAs**.** Pearson correlation coefficient and *p*-value are shown above each plot. *n* = 16 subjects (8 HIV-positive and 8 HIV-negative). DHA, docosahexaenoate (22:6n3); n3 DPA, docosapentaenoate (22:5n3); n6 DPA, docosapentaenoate (22:5n6) and EPA, eicosapentaenoate (20:5n3).

**Additional file 2: Supplementary Table S1** - Biotype counts of different RNA species identified by RNA-sequencing of plasma EV small RNAs from HIV-positive and HIV-negative subjects in the test cohort.

**Additional file 3: Supplementary Table S2** - Read counts (normalized RPM) of all mapped mature plasma EV miRNAs in the test cohort.

**Additional file 4: Supplementary Table S3** - Differential expression analysis of top 70 protein coding reads showing fold change between HIV-positive (*n* = 8) versus HIV-negative (*n* = 12) subjects in the test cohort. 70 genes were selected from a total of 14,500 genes by filtering for protein coding sequences with counts > 10 in at least 25% samples, and not identified in a blank sample from an independent experiment.

**Additional file 5: Supplementary Table S4** - Over-representation analysis of differentially expressed plasma EV-miRNAs in the validation cohort using miEAA (miRNA Enrichment Analysis and Annotation) tool.

**Additional file 6: Supplementary Table S5** - Predicted and validated target genes of 6 differentially expressed plasma EV-miRNAs in the validation cohort. Shown are 61 target genes selected based on the rule: 1) present in both miRDIP and miRWalk predicted target lists; OR 2) present in either miRDIP or miRWalk predicted target lists and experimentally validated (miRTarbase).

**Additional file 7: Supplementary Table S6** - Enriched pathway terms of networks associated with 61 predicted and validated target genes of 6 miRNAs upregulated in the validation cohort using ClueGo software (.xlsx)

**Additional file 8: Supplementary Table S7** - Levels of plasma metabolites associated with oxidative stress, polyunsaturated fatty acid (PUFA), and tryptophan/kynurenine metabolism in HIV-negative (*n* = 8) and HIV-positive (*n* = 8) subjects in the validation cohort.

**Additional file 9: Supplementary Figure S6** – Full-length images of blots shown in Fig. 2c.

## Data Availability

All data generated or analyzed during this study are included in this published article (and its Supplementary Data files) or available from the corresponding author on reasonable request.

## Abbreviations

ART: Antiretroviral therapy
ALT: Alanine aminotransferase
CSF: Cerebrospinal fluid
EV: Extracellular vesicles
FDR: False discovery rate
HIV: Human immunodeficiency virus type I
K:T ratio: Kynurenine: Tryptophan ratio
lincRNA: Long intergenic noncoding RNA
miRNA: MicroRNA
mRNA: Messenger RNA
NTA: Nanoparticle tracking analysis
ORA: Over-representation analysis
PCA: Principal component analysis
piRNA: Piwi-interacting RNA
rRNA: Ribosomal RNA
ROS: Reactive oxygen species
snoRNA: Small nucleolar RNA
snRNA: Small nuclear RNA
TEM: Transmission electron microscopy; tRNA: transfer RNA
