# Supplementary material for: Small RNA sequencing of extracellular vesicles identifies circulating miRNAs related to inflammation and oxidative stress in HIV patients
Source: BMC Immunol. 2020 Nov 11;21:57. doi: 10.1186/s12865-020-00386-5 (PMC7656686; doi:10.1186/s12865-020-00386-5)
Supplement: Supplementary file 1 — Additional file 1: Supplementary Figure S1: Comparison of total read counts for each RNA biotype in HIV-positive (n = 12) versus HIV-negative (n = 12) subjects. Supplementary Figure S2. Principal component analysis of the top 50 miRNAs identified by small RNA sequencing of plasma EV RNAs from HIV-positive (n = 12) and HIV-negative (n = 12) subjects. Three HIV-negative outliers (circled in green) were excluded from downstream differential expression analysis. Supplementary Figure S3. Plasma EV isolation and purification to exclude extravesicular RNAs. EV fractions were isolated from pooled plasma of healthy control subjects (n = 3) using the PureExo kit. Defibrinated plasma was either untreated, or treated with RNAse A, or with Proteinase-K followed by RNAse A, to eliminate extravesicular RNAs. TEM (top), particle size distribution (bottom left), particle concentration (bottom middle), and immunoblotting for exosome markers and ApoA1 (bottom right) are shown for each treatment condition. Supplementary Figure S4. Comparison of Cq values of miRNAs in plasma EVs of HIV-positive and HIV-negative subjects in the validation cohort, stratified by cocaine use. Mean and SEM are shown. Significance was calculated using Mann Whitney test. (n = 8 HIV-positive and n = 8 HIV-negative subjects). Supplementary Figure S5: Scatter plots showing inverse relationships between PUFA metabolites and EV-associated miRNAs. Pearson correlation coefficient and p-value are shown above each plot. n = 16 subjects (8 HIV-positive and 8 HIV-negative). DHA, docosahexaenoate (22:6n3); n3 DPA, docosapentaenoate (22:5n3); n6 DPA, docosapentaenoate (22:5n6) and EPA, eicosapentaenoate (20:5n3). [file 12865_2020_386_MOESM1_ESM.docx]

**Small RNA Sequencing of Extracellular Vesicles Identifies Circulating MiRNAs Related to**

**Inflammation and Oxidative Stress in HIV Patients**

Sukrutha Chettimada^1^, David R. Lorenz^1^, Vikas Misra^1^, Steven M. Wolinsky^2^, and Dana Gabuzda^1,3^

**Affiliations:**

^1^Dana-Farber Cancer Institute, Department of Cancer Immunology and Virology, Boston, MA

^2^Division of Infectious Diseases, Department of Medicine, Northwestern University Feinberg School of Medicine, Chicago, IL, USA

^3^Department of Neurology, Harvard Medical School, Boston MA

**Corresponding author:**

Dr. Dana Gabuzda, email: [dana_gabuzda@dfci.harvard.edu](mailto:dana_gabuzda@dfci.harvard.edu)

This file includes:

1. Supplementary Figure S1
2. Supplementary Figure S2
3. Supplementary Figure S3
4. Supplementary Figure S4
5. Supplementary Figure S5

**Supplementary Figure S1:** Comparison of total read counts for each RNA biotype in HIV-positive (n=12) versus HIV-negative (n=12) subjects


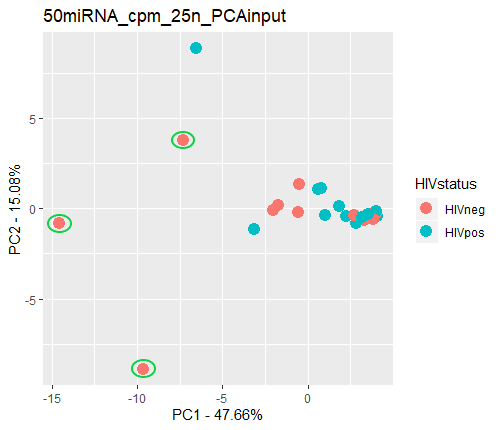


**Supplementary Figure S2**. Principal component analysis of the top 50 miRNAs identified by small RNA sequencing of plasma EV RNAs from HIV-positive (n=12) and HIV-negative (n=12) subjects. Three HIV-negative outliers (circled in green) were excluded from downstream differential expression analysis.

**Supplementary Figure S3**. Plasma EV isolation and purification to exclude extravesicular RNAs. EV fractions were isolated from pooled plasma of healthy control subjects (n=3) using the PureExo kit. Defibrinated plasma was either untreated, or treated with RNAse A, or with Proteinase-K followed by RNAse A, to eliminate extravesicular RNAs. TEM (top), particle size distribution (bottom left), particle concentration (bottom middle), and immunoblotting for exosome markers and ApoA1 (bottom right) are shown for each treatment condition.

**+RNAseA**

**+ Proteinase-K +RNAse**


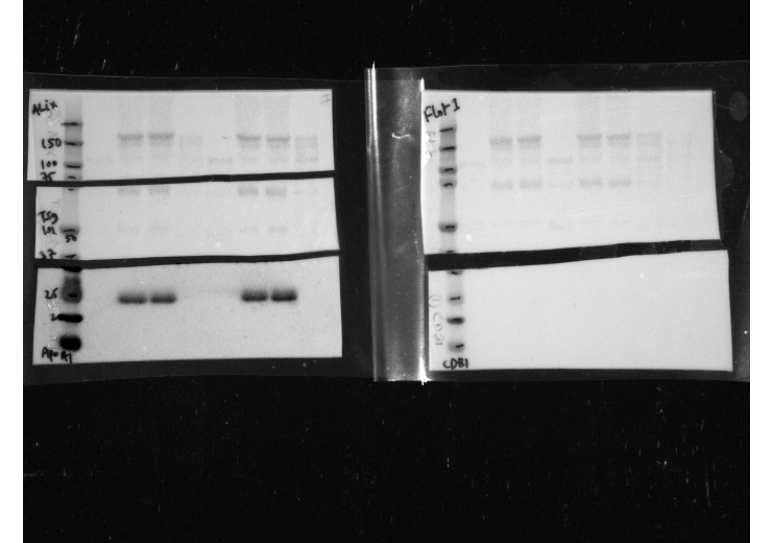

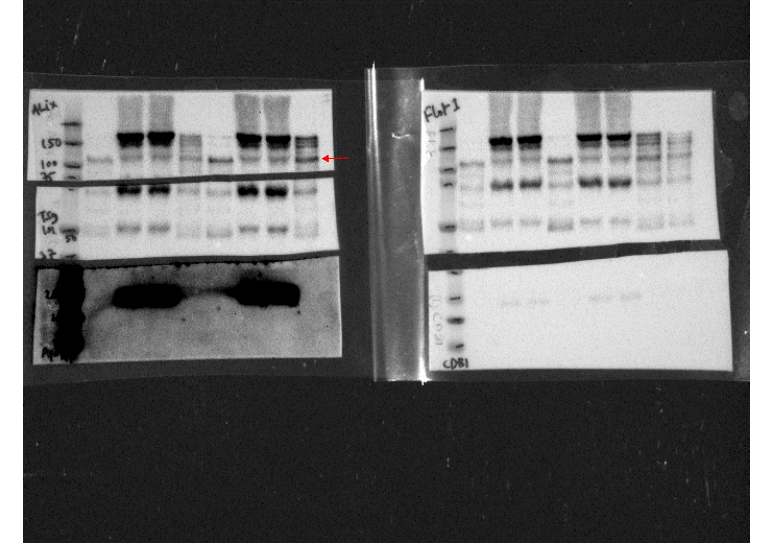

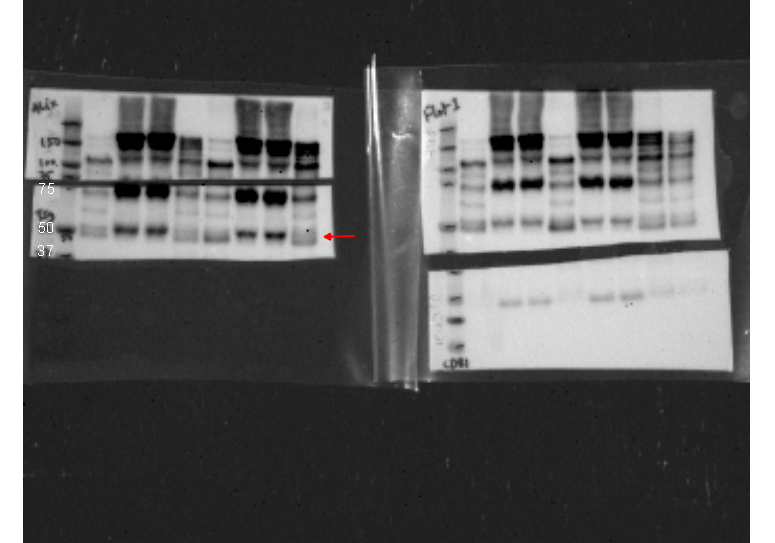

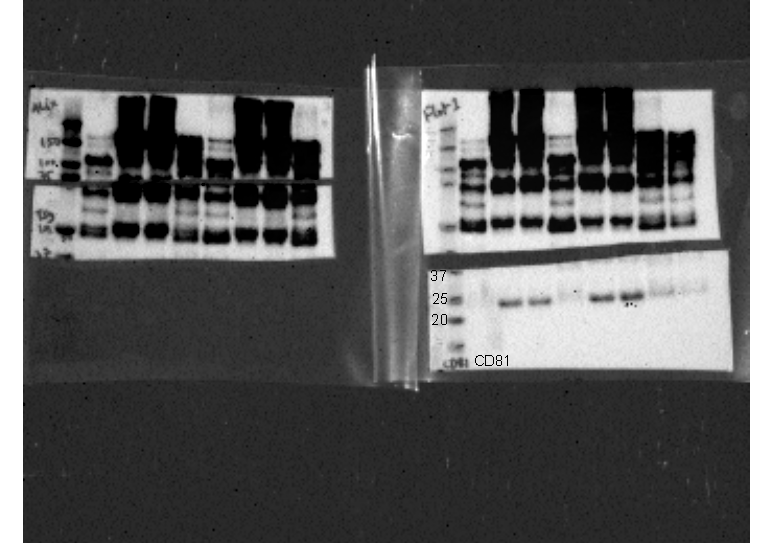

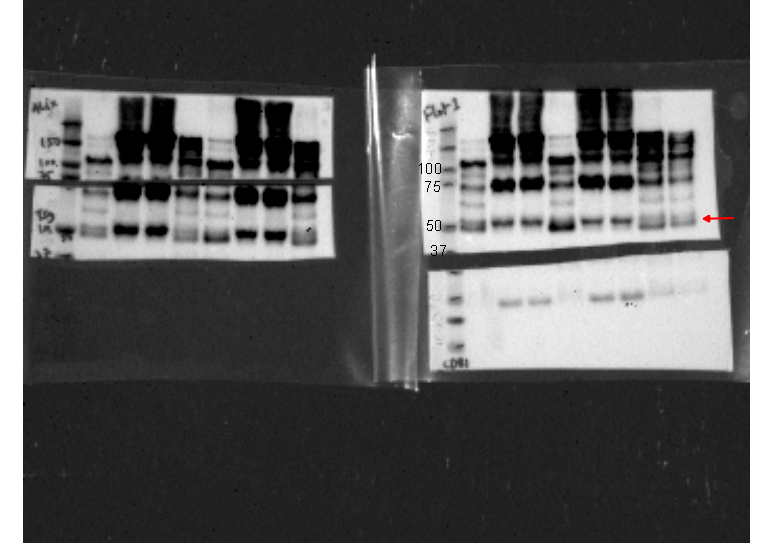


**Flotillin-1** (48 kDa)

**CD81** (28 kDa)

**Alix** (95 kDa)

**Tsg101** (46 kDa)

**ApoA1** (30 kDa)

**Proteinase-K** **- - +**

**RNAse A - + +**

**Untreated**


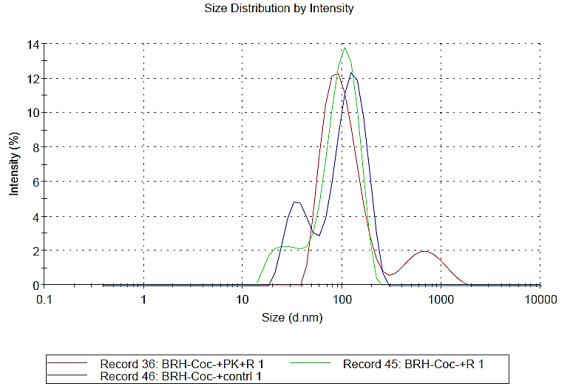


Control

RNAseA

P-K+RNAseA

**Particle concentration**

**Particle size distribution**


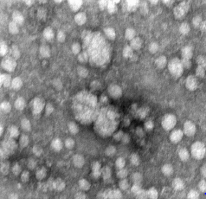

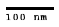

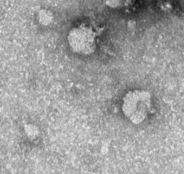

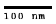

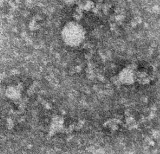

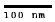

**Supplementary Figure S4**. Comparison of Cq values of miRNAs in plasma EVs of HIV-positive and HIV-negative subjects in the validation cohort, stratified by cocaine use. Mean and SEM are shown. Significance was calculated using Mann Whitney test. (n=8 HIV-positive and n=8 HIV-negative subjects)

**Supplementary Figure S5:** Scatter plots showing inverse relationships between PUFA metabolites and EV-associated miRNAs**.** Pearson correlation coefficient and p-value are shown above each plot. n= 16 subjects (8 HIV-positive and 8 HIV-negative). DHA, docosahexaenoate (22:6n3); n3 DPA, docosapentaenoate (22:5n3); n6 DPA, docosapentaenoate (22:5n6) and EPA, eicosapentaenoate (20:5n3)
