# Supplementary figures and images for: Small RNA sequencing of extracellular vesicles identifies circulating miRNAs related to inflammation and oxidative stress in HIV patients
Source: BMC Immunol. 2020 Nov 11;21:57. doi: 10.1186/s12865-020-00386-5 (PMC7656686; doi:10.1186/s12865-020-00386-5)

## Slide 1
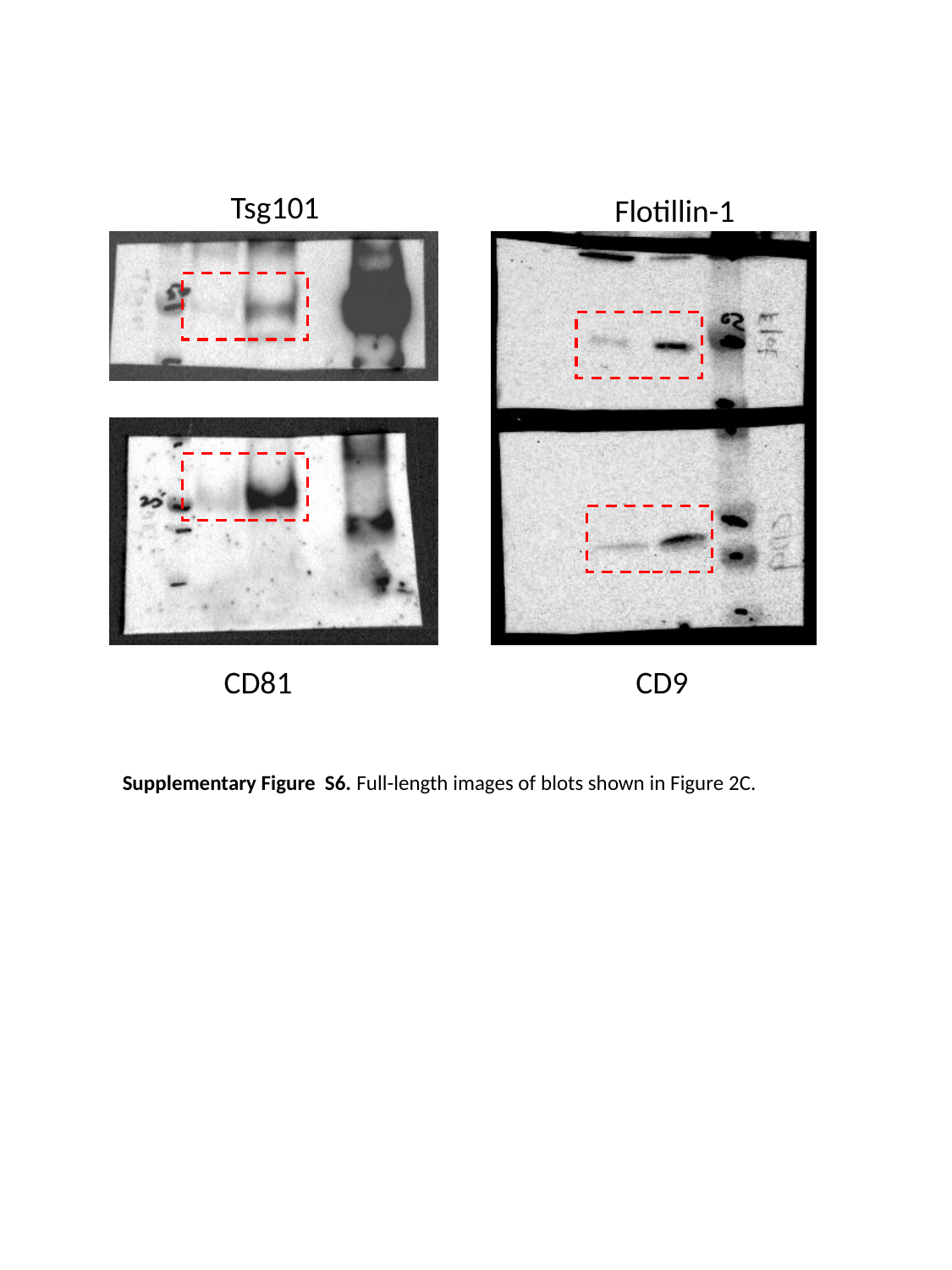

Tsg101
Flotillin-1
CD81
CD9
Supplementary Figure S6. Full-length images of blots shown in Figure 2C.

Supplement: Supplementary file 9 — Additional file 9: Supplementary Figure S6 – Full-length images of blots shown in Fig. 2c. [file 12865_2020_386_MOESM9_ESM.pptx]
